# Efficacy and safety of the combination of propofol and S(+)-ketamine for procedural sedation in pediatric patients undergoing totally implantable venous access port implantation: A prospective randomized controlled study

**DOI:** 10.3389/fped.2022.974917

**Published:** 2022-08-17

**Authors:** Yingjun Zhang, Chaopeng Ou, Xiaohui Bai, Jielan Lai, Wan Huang, Handong Ouyang

**Affiliations:** ^1^State Key Laboratory of Oncology in Southern China, Collaborative Innovation Center for Cancer Medicine, Department of Anesthesiology, Sun Yat-sen University Cancer Center, Guangzhou, China; ^2^Guangdong Provincial Key Laboratory of Malignant Tumor Epigenetics and Gene Regulation, Department of Anesthesiology, Sun Yat-sen Memorial Hospital, Sun Yat-sen University, Guangzhou, China

**Keywords:** pediatric, procedural sedation, propofol, S(+)-ketamine, totally implantable venous access port

## Abstract

**Background:**

Totally implantable venous access port (TIVAP) implantation is usually performed under general anesthesia with endotracheal intubation in children. Procedural sedation without endotracheal intubation has been applied to minor pediatric surgeries like central venous catheter insertion. To explore a more efficient and less invasive anesthesia mode to implant TIVAPs for children, we aimed to evaluate the efficacy and safety of procedural sedation using propofol and S(+)-ketamine compared with general anesthesia.

**Methods:**

Sixty-six patients aged 6 months to 10 years undergoing TIVAP implantation were randomly allocated to two groups. Patients under procedural sedation [S(+)-ketamine-propofol (sketofol) group] were given target-controlled infusion of propofol 4 μg/ml using the Paedfusor model and S(+)-ketamine 0.5 mg/kg as induction, and had target-controlled infusion of propofol 3–4 μg/ml as maintenance. Patients in sketofol group received medium-flow oxygen inhalation through facemasks during surgery. Patients under general anesthesia (control group) were given propofol 2 mg/kg, cisatracurium 0.2 mg/kg, fentanyl 3 μg/kg as induction, and sevoflurane 0.8 minimum alveolar concentration as maintenance after endotracheal intubation. Primary outcome was the postoperative emergence agitation evaluated 5 min after awakening.

**Results:**

Postoperative emergence agitation evaluated 5 min after awakening was lower in sketofol group versus control group [1.0 (0.5, 1.0) vs. 3.0 (2.0, 4.0); median difference (95% CI): 2.0 (1.0, 2.0); *P* < 0.001]. Time to awakening was significantly lower in sketofol group versus control group [15.0 (5.0, 23.0) vs. 26.0 (20.5, 37.5); median difference (95% CI): 11.0 (7.0, 19.0); *P* < 0.001], as well as time to discharge from post anesthesia care unit [35.0 (24.0, 45.0) vs. 45.0 (37.5, 59.5); median difference (95% CI): 10.0 (10.0, 23.0); *P* < 0.001]. Postoperative complications or adverse events were not reported in sketofol group.

**Conclusions:**

Compared to general anesthesia with endotracheal intubation, procedural sedation using propofol and S(+)-ketamine improves the postoperative emergence agitation right after the recovery of consciousness, and has advantage in shortening anesthetic recovery time for pediatric patients undergoing TIVAP implantation.

## Introduction

A large number of patients with malignant tumor are in need of long-term infusion, aiming for infusion of highly concentrated chemotherapy or long-term parenteral nutrition solution. Central venous catheters and totally implantable venous access ports have been mainstream methods of establishing venous access for patients with malignancy, especially for pediatric patients (1). The totally implantable venous access port, which is known as TIVAP, has been invaluable for oncology patients since 1980s (2). TIVAPs can be retained for a longer period of time. Meanwhile, it could reduce the pain and difficulty of repeated peripheral venous punctures, making it ideal for the intravenous treatment in pediatric patients with malignancy (3). Furthermore, studies have demonstrated that infection risk is lower in TIVAPs than tunneled central venous catheters (4).

Since most pediatric patients, especially those aged below ten, cannot cooperate throughout the whole process due to their immaturity, the surgery to implant TIVAPs has been performed under general anesthesia (5). However, the risk of general anesthesia in pediatric patients is an important issue to be reckoned with. Clinical statistics have demonstrated that pediatric patients might suffer from higher risk of bronchospasm, laryngospasm, hypoxia or respiratory obstruction after endotracheal intubation (6). Sevoflurane, the widely used inhalation anesthetics in general anesthesia, has been reported to be more likely to cause laryngospasm and postoperative delirium than propofol in general anesthesia (7, 8). In pediatric clinical practice, procedures like central venous catheter insertion, bone marrow aspiration, biopsy or magnetic resonance imaging would be done under procedural sedation, utilizing propofol or benzodiazepines combined with or without opioid or ketamine (9, 10).

S(+)-ketamine is widely used in pediatric patients because of its sedative, anesthetic and amnesic effects (11, 12). Studies have demonstrated that S(+)-ketamine combined with propofol or benzodiazepines could be used as a sedative solution for manipulation of forearm fractures, dentistry surgery or some other short-term procedures in children, fulfilling the goal of proper sedation while maintaining the spontaneous breathing (13, 14). Generally speaking, the overall procedure to establish TIVAPs would take up 20–30 min. Since it is not a very complicated and long-lasting procedure, it is theoretically possible to perform it under procedural sedation. Yet there is not much evidence of implanting TIVAPs under procedural sedation in pediatric patients (15). As patients under procedural sedation do not require endotracheal intubation, extubation is not needed and patients can feel more comfortable during the recovery period. To explore a more efficient and optimal anesthesia mode to implant TIVAPs for pediatric patients, we carried out the study to compare the effect of procedural sedation using S(+)-ketamine and propofol without endotracheal intubation and general anesthesia with endotracheal intubation.

## Methods

### Ethics

In this randomized controlled study, children aged from 6 months to 10 years and scheduled for TIVAP implantation were randomly assigned to a S(+)-ketamine-propofol (sketofol) group or a control group. The study was approved by the institutional research ethics committee of Sun Yat-sen University Cancer Center and was carried out in this hospital. The study was registered at www.chictr.org.cn (Number: ChiCTR2200060384). Parental or guardian's written informed consents were obtained before enrollment.

### Inclusion and exclusion

The patients enrolled were aged between 6 months and 10 years, male or female, with American Society of Anesthesiologists (ASA) grade I-II, and were to receive TIVAP implantation. Exclusion criteria included patients with severe malnutrition, moderate or severe anemia, acute respiratory infection within 2 weeks, ongoing fever with temperature above 38 °C, allergy or tolerance history with S(+)-ketamine or propofol, or under treatment with sedative or analgesic drugs. Patients with ASA grade III-V were also excluded. If the patient was lost to follow-up, the case would be withdrawn from the study.

### Randomization and data collection

The randomization of the study (1:1) was accomplished through internet-based randomization software (http://www.randomization.com). Intraoperative data were collected by investigators and postoperative data were collected by nurses and investigators.

### Study protocol

Parental or guardian's consents were performed in the preoperative area followed by baseline measurement. Patient's gender, age, height, weight, previous medical history and allergic history were confirmed at the same time. The parental separation anxiety scale (PSAS) was evaluated before the patients entered the operation room (Table 1).

**Table 1:** Evaluation criteria for parental separation anxiety scale, emergence agitation.

**Performance**	**Scale**
**Parental separation anxiety scale**	
Easy separation	1
Whimpers, but is easily reassured, not clinging	2
Cries and can't be easily reassured, but not clinging	3
Crying and clinging to parents	4
**Emergence agitation**	
Asleep	0
Calm	1
Crying, but can be consoled	2
Crying, but can't be consoled	3
Agitated and trashing around	4

Investigators confirmed the result of randomization. The anesthesia strategy of each group was as follows. Patients in the sketofol group were given target-controlled infusion of propofol 4 μg/ml using the Paedfusor model (Alaris PK Syringe Pump, Carefusion, Somerset, England) and S(+)-ketamine 0.5 mg/kg as induction, and had target-controlled infusion of propofol 3–4 μg/ml as maintenance. All patients of the sketofol group received medium-flow oxygen inhalation through facemasks during the whole procedure. Patients in control group were given propofol 2 mg/kg, cisatracurium 0.2 mg/kg, and fentanyl 3 μg/kg as induction, and inhaled sevoflurane with the goal of reaching 0.8 minimum alveolar concentration (MAC) as maintenance after endotracheal intubation. All subjects in both groups had infiltration anesthesia with 1% lidocaine around the spot of the TIVAP emplacement before incision of skin. All anesthetics would be withdrawn in both group when the surgeon began to suture the skin. Blood pressure, heart rate and oxygen saturation were routinely recorded every 5 min after the patients entered the operation room. Investigators recorded vital signs every 5 min in post anesthesia care unit (PACU). The recovery criteria were complete recovery of spontaneous breathing and eye opening recovery in control group, and eye opening recovery in sketofol group. Time to awakening was defined as the period from end of surgery to moment of reaching recovery criteria. The postoperative emergence agitation scale (Table 1) was evaluated 5 min after awakening by an individual investigator who was not aware of the result of randomization and allocation. Investigators followed up on postoperative complications like hypoxia, vomiting within 2 days after surgery.

### Measurements

The primary outcome of the study was the postoperative emergence agitation evaluated 5 min after awakening. The secondary outcomes were body movement during operation, hypoxia during surgery, surgery length, anesthesia length, time to awakening, time to discharge from PACU, adverse events and postoperative complications including vomiting, hypoxia.

### Sample size calculation

The primary outcome of the study was the postoperative emergence agitation evaluated 5 min after awakening. Software PASS (PASS 15 Power Analysis and Sample Size Software 2017, NCSS, LLC. Kaysville, Utah, USA, ncss.com/software/pass) was used to estimate the sample size. Based on our previous clinical practice, the mean postoperative emergence agitation evaluated 5 min after awakening of children receiving TIVAP implantation under general anesthesia was 2.96, with a standard deviation of 1.05. We assumed that procedural sedation in combination of propofol and S(+)-ketamine would improve the postoperative emergence agitation evaluated 5 min after awakening by reducing at least 30%. A sample size of 31 patients per group was estimated using power of 90%, and significance level of 5%. To allow for potential dropout of 5%, 66 patients were recruited.

### Statistical analysis

Data were analyzed using SPSS (SPSS version 28.0, IBM, USA). Normal distribution of continuous variables was first evaluated using the Shapiro-Wilk test. Data were expressed as frequencies for categorical variables and means (standard deviations) or medians (inter-quartile ranges) for continuous variables, as appropriate. All data were compared between the groups using the χ^2^ test or Fisher's exact test for categorical variables and Student's *t*-test or Mann-Whitney test for continuous variables, as appropriate. A *P*-value < 0.05 was considered statistically significant.

## Results

**Figure 1:**
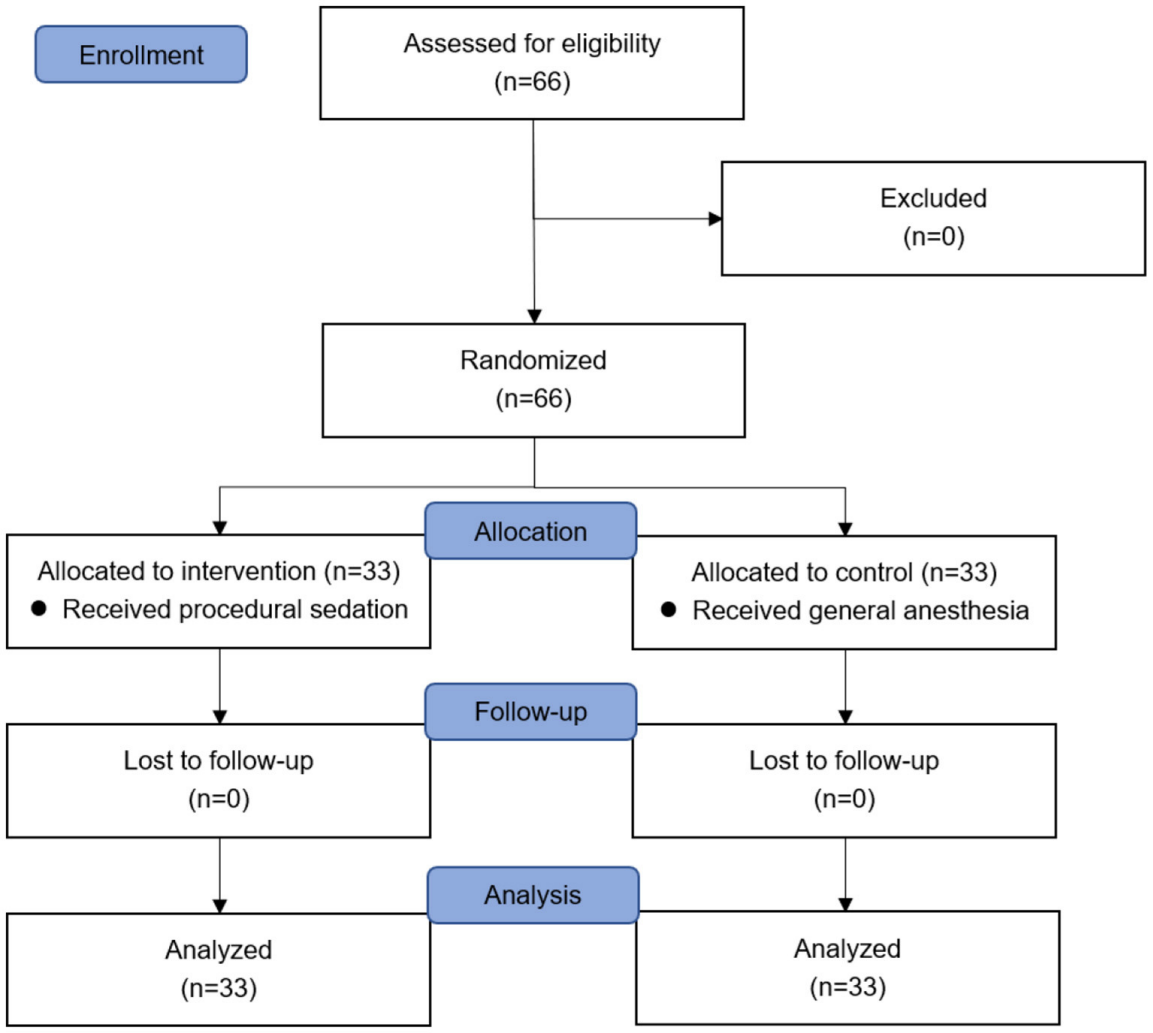
CONSORT study flow diagram.

Following the inclusion and exclusion criteria, 66 patients were assessed for eligibility and recruited to the study. Sixty-six patients were randomized to two equal groups (Figure 1). No subjects were lost to follow-up. Patient characteristics were comparable between two groups (Table 2). Baseline variables were not substantially different between two groups.

**Table 2:** Subject characteristic data.

	**Sketofol**	**Control**	***P*-value**
Age, years	4.51 ± 3.13	4.23 ± 2.82	0.708
Age range, *n* (%)			0.482
≥ 0.5 and <1 years	2 (6.1)	3 (9.1)	
≥ 1 and <3 years	12 (36.4)	10 (30.3)	
≥ 3 and <7 years	8 (24.2)	13 (39.4)	
≥ 7 and ≤ 10 years	11 (33.3)	7 (21.2)	
Gender			0.438
Male	20 (60.6)	23 (69.7)	
Female	13 (39.4)	10 (39.4)	
Height	104.46 ± 25.37	102.23 ± 22.25	0.706
Weight	18.61 ± 10.69	17.82 ± 9.04	0.745
Weight range, *n* (%)			1.000
<10 kg	7 (21.2)	7 (21.2)	
≥10 kg	26 (78.8)	26 (78.8)	
ASA class, *n* (%)			0.614
I	12 (36.4)	14 (42.4)	
II	21 (63.6)	19 (57.6)	
Preoperative PSAS	2.36 ± 1.32	2.70 ± 1.24	0.294

*Data are expressed as mean± standard deviation, or number (%)*.

*PSAS, parental separation anxiety scale*.

Results of the primary outcomes are shown in Table 3. Postoperative emergence agitation evaluated 5 min after awakening was lower in the sketofol group than in the control group [1.0 (0.5, 1.0) vs. 3.0 (2.0, 4.0); median difference (95% CI): 2.0 (1.0, 2.0); *P* < 0.001].

**Table 3:** Primary outcome: postoperative emergence agitation evaluated 5 min after awakening.

	**Sketofol**	**Control**	**Median difference (95% CI)**	***P*-value**
Postoperative emergence agitation	1.0 (0.5, 1.0)	3.0 (2.0, 4.0)	2.0 (1.0, 2.0)	<0.001

*Data are expressed as median (interquartile range)*.

*CI, confidence interval*.

Table 4 demonstrates the comparison of mean blood pressures and heart rates at specific time-points between two groups. The baseline mean blood pressure and heart rate were not significantly different between the two groups. There was no significant difference between two groups during the surgery process and the period of unconsciousness at PACU. However, mean blood pressure was higher in patients of control group after extubation than in patients of sketofol group right after consciousness recovery.

**Table 4:** Mean blood pressures and heart rates at different time-points.

	**Sketofol**	**Control**	**95% CI of differences**	***P*-value**
**Preoperative**				
Mean blood pressure	69.98 ± 12.80	74.53 ± 12.95	−1.79 to 10.88	0.157
Heart rate	105.29 ± 14.58	108.64 ± 20.52	−5.60 to 11.91	0.475
**Beginning of surgery**				
Mean blood pressure	65.20 ± 10.99	71.11 ± 14.23	−0.34 to 12.16	0.064
Heart rate	101.76 ± 16.61	103.00 ± 20.94	−8.05 to 10.54	0.790
**10 min after surgery began**				
Mean blood pressure	65.09 ± 11.60	70.20 ± 14.65	−1.39 to 11.61	0.121
Heart rate	100.58 ± 14.22	99.33 ± 24.10	−10.97 to 8.49	0.800
**Patients transferred to PACU**				
Mean blood pressure	75.46 ± 17.31	80.62 ± 16.35	−3.13 to 13.43	0.219
Heart rate	102.76 ± 19.38	98.85 ± 18.94	−13.33 to 5.51	0.410
**After extubation / consciousness recovery**				
Mean blood pressure	80.19 ± 15.23	95.21 ± 14.68	7.67 to 22.38	<0.001
Heart rate	105.06 ± 21.12	114.36 ± 27.40	−2.73 to 21.33	0.127

*Data are expressed as mean ± standard deviation*.

*PACU, post anesthesia care unit; CI, confidence interval*.

Table 5 presents the secondary outcomes of the study. Ratio of unconscious body movement was higher in sketofol group vs. control group (9.1% vs. 0%, *P* = 0.076), which was not significantly different. All unconscious body movements happened at the time when infiltration anesthesia was performed. However, this kind of body movement was mild and transient, and did not affect the surgery. Hypoxia during surgery, surgery length and anesthesia length were not significantly different between the two groups. Time to awakening was significantly lower in the sketofol group vs. control group [15.0 (5.0, 23.0) vs. 26.0 (20.5, 37.5); median difference (95% CI): 11.0 (7.0, 19.0); *P* < 0.001]. Time to discharge from PACU was significantly lower in the sketofol group vs. control group [35.0 (24.0, 45.0) vs. 45.0 (37.5, 59.5); median difference (95% CI): 10.0 (10.0, 23.0); *P* < 0.001].

**Table 5:** Secondary outcomes: body movement during operation, hypoxia during surgery, surgery length, anesthesia length, time to awakening, time to discharge from PACU, postoperative vomiting and postoperative hypoxia.

	**Sketofol**	**Control**	**Median difference (95% CI)**	***P*-value**
Body movement during operation, *n* (%)	3 (9.1)	0 (0)		0.076
Hypoxia during surgery, *n* (%)	0 (0)	0 (0)		1
Surgery length (min)	25.0 (20.0, 30.0)	23.0 (20.0, 27.5)	−2.0 (−4.0, 2.0)	0.312
Anesthesia length (min)	35.0 (29.5, 38.5)	35.0 (31.0, 42.5)	0.0 (−2.0, 6.0)	0.277
Time to awakening (min)	15.0 (5.0, 23.0)	26.0 (20.5, 37.5)	11.0 (7.0, 19.0)	<0.001
Time to discharge from PACU (min)	35.0 (24.0, 45.0)	45.0 (37.5, 59.5)	10.0 (10.0, 23.0)	<0.001
Transient hypoxia at PACU, *n* (%)	0, (0)	7, (21.2)		0.005
Consistent postoperative hypoxia, *n* (%)	0, (0)	0, (0)		1
Postoperative vomiting, *n* (%)	0, (0)	0, (0)		1

*Data are expressed as number (%) or median (interquartile range)*.

*PACU, post anesthesia care unit; CI, confidence interval*.

As for postoperative complications, there was no case from either group suffering from vomiting, or consistent postoperative hypoxia. 21.2% of patients in control group went through transient hypoxia after extubation at PACU, while all patients in sketofol group remained SPO_2_ ≥ 95% at PACU throughout the whole time.

## Discussion

In this prospective randomized controlled trial, we found that patients under procedural sedation using propofol and S(+)-ketamine without endotracheal intubation had lower postoperative emergence agitation evaluated 5 min after awakening, compared to general anesthesia with endotracheal intubation. Meanwhile, time to awakening was shorter in patients under procedural sedation using propofol and S(+)-ketamine than those under general anesthesia.

Though TIVAP implantation is not a complex or time-consuming procedure, most of the pediatric patients are not mature enough to tolerate the procedure simply under local anesthesia. In our clinical practice, TIVAP implantation was performed under general anesthesia with tracheal intubation in pediatric patients, especially in those aged below 10. A systematic review done by Ng et al. (16) demonstrated a decreased incidence in emergence delirium vs. placebo when ketamine was given intraoperatively. It is consistent with our results, as patients in sketofol group had lower emergence agitation scale than those in control group. There is a risk of damaging tracheal or laryngeal mucosa when endotracheal intubation or laryngeal mask airway is used. During the process of TIVAP implantation, insufficient ventilation may happen using laryngeal mask airway as patients' heads and necks need to be leaned to one side and surgeons have to perform the surgery around the necks. A prospective cohort study showed that sevoflurane increased incidence of laryngospasm compared with propofol and the incidence of all perioperative respiratory adverse events, particularly laryngospasm, was increased after direct stimulation of the upper airways by laryngeal mask airway or tracheal tube (6). In this study, 21.2% of patients with endotracheal intubation suffered from transient hypoxia after extubation at PACU, while all patients under procedural sedation remained SPO_2_ above 95% throughout the recovery time.

It is of crucial importance to ensure airway patency and maintain spontaneous breathing during procedural sedation. Adverse events of propofol include hypotension and respiratory depression to some extent. Ketamine, a phencyclidine derivative, is known as its effect of “dissociative anesthesia.” In high doses, ketamine produces anesthesia and analgesia and in low doses it acts as an analgesic drug (17). These effects are mainly mediated by non-competitive antagonism of the N-methyl-D-aspartate (NMDA) receptors in the central nervous system. And ketamine has effects on muscle function *via* other channels, which specifically causes bronchodilation but keeps the gag reflex intact and rarely requires intubation. S(+)-ketamine has a higher affinity for the NMDA receptor than the racemic compound, and thus lower doses are required to produce anesthesia and analgesia. Meanwhile, the psychotomimetic adverse effects are dose dependent. Studies demonstrated that S-ketamine is two and four times more potent as an anesthetic and analgesic than the racemate and R(-)-isomer, respectively (18, 19). Therefore, S(+)-ketamine has stronger efficacy of anesthesia and analgesia, while it is less likely to produce psychiatric symptoms (20). Adverse respiratory events such as laryngospasm are rare, at a reported of 0.4–0.7% (21). S(+)-ketamine produces hemodynamically stable anesthesia *via* central sympathetic stimulation without affecting respiratory function, making the combination of propofol and S(+)-ketamine ideal for procedural sedation (22). A study done by Shetabi et al. (23) reported that the drug combination of propofol and ketamine was suggested to be used in patients under chemotherapy while performing placement and removal of port catheter.

In this trial, time to awakening was shorter in patients under procedural sedation than in those under general anesthesia. At the same time, patients under procedural sedation had lower postoperative emergence agitation right after the recovery of consciousness and required shorter length of stay at PACU. It was probably because lower dosage and fewer types of anesthetics were used. Throughout the process of surgery, the mean blood pressures and heart rates were not significantly different between two groups, which indicates that intravenous administration of propofol and S(+)-ketamine is qualified to produce a sufficient depth of sedation. In the recovery period at PACU, the mean blood pressures and heart rates in sketofol group were more consistent with the baseline level without the existence of tracheal catheter and stimulation of extubation. Since muscle relaxant was not applied in the cases of procedural sedation, a small portion of patients in sketofol group appeared to have unconscious body movements when infiltration anesthesia with lidocaine was performed in the specific spot of TIVAP emplacement. However, this kind of body movement was mild and transient, and did not affect the surgery.

Several limitations of this study should be noted. First, the sketofol group received just intravenous anesthetics while the control group received intravenous and volatile anesthetics. As inhalation anesthesia is much more commonly used in China than total intravenous anesthesia, sevoflurane was used in the control group aiming at making the result of this study closer to reality. If only intravenous anesthetics are applied in both groups, the results will be more convincing, Second, there was no monitoring item to represent the depth of anesthesia in the study. Monitoring index like narcotrend provide evidence that patients in both groups are under proper depth of anesthesia, which minimize the bias between two groups. Third, children differ greatly in maturity and cooperation due to their age. It is more appropriate to narrow down the range of age and expand the scale of subjects. Fourth, the scale of the study was small. Larger number of participants are needed for more convincing results.

## Conclusions

Compared to general anesthesia with endotracheal intubation, procedural sedation using propofol and S(+)-ketamine has an advantage in improving postoperative emergence agitation right after the recovery of consciousness for pediatric patients undergoing TIVAP implantation. Pediatric patients under procedural sedation with propofol and S(+)-ketamine require shorter time to awakening and length of stay at PACU vs. general anesthesia.

## Data availability statement

The raw data supporting the conclusions of this article will be made available by the authors, without undue reservation.

## Ethics statement

The studies involving human participants were reviewed and approved by the Institutional Research Ethics Committee of Sun Yat-sen University Cancer Center. Written informed consent to participate in this study was provided by the participants' legal guardian/next of kin.

## Author contributions

YZ, CO, HO, and WH devised and wrote the study protocol. YZ, CO, and HO contributed to the data analysis and production of the final manuscript. All authors took part in conducting the study and collecting data. All authors contributed to the article and approved the submitted version.

## Funding

This work was supported by the HO's Clinical Medical Scientists Project of Sun Yat-sen University Cancer Center (PT09090101) and Medical Scientific Research Foundation of Guangdong Province, China (Grant Number: C2020058) and Scientific Research Fund of Traditional Chinese Medicine Bureau of Guangdong Province (No. 20211099).

## Conflict of interest

The authors declare that the research was conducted in the absence of any commercial or financial relationships that could be construed as a potential conflict of interest.

## Publisher's note

All claims expressed in this article are solely those of the authors and do not necessarily represent those of their affiliated organizations, or those of the publisher, the editors and the reviewers. Any product that may be evaluated in this article, or claim that may be made by its manufacturer, is not guaranteed or endorsed by the publisher.
